# Prurigo strophulus: Epidemiological, clinical aspects and environmental factors among children in Yaoundé, Cameroon (Sub‐Saharan Africa)

**DOI:** 10.1002/ski2.38

**Published:** 2021-05-05

**Authors:** E. A. Kouotou, U. Nguena Feungue, A. Engolo Fandio, D. N. Tounouga, E. C. Ndjitoyap Ndam

**Affiliations:** ^1^ Department of Internal Medicine and Specialties Faculty of Medicine and Biomedical Sciences University of Yaoundé I Yaoundé Cameroon; ^2^ Yaoundé University Teaching Hospital Yaoundé Cameroon; ^3^ University Hospital of Treichville Abidjan Ivory Coast; ^4^ Lafe‐Baleng Divisional Health Centre Bafoussam Cameroon

## Abstract

**Background:**

Prurigo Strophulus (PS) is a chronic and recurrent inflammatory disease caused by a hypersensitivity reaction to arthropod bites. The objective of this study was to determine the prevalence of PS and its clinical characteristic in our context.

**Methods:**

We performed a cross‐sectional study during February to May 2017 in Dermatology's units of six hospitals in Yaoundé. Children with clinical signs of PS were included. A questionnaire was administered for data collection.

**Results:**

A total of 112 children (62 boys and 50 girls) were included in the study, with a median age of 2 years; with range varied from 5 months to 16 years. The prevalence of PS was 5.4%. The most represented age group was 0–5 years (78.6%). PS lesions were most often located in exposed areas of body such as lower limbs (101/112; 90.2%), upper limbs (85/112; 75.9%) and face (19.6%). Papule‐vesicle (87.5%) was the predominant type of lesions.

**Conclusion:**

PS is a common disease in Yaoundé (Cameroon). Papule‐vesicle lesions are the most frequent signs. It is usually found in exposure areas of body.

## Graphical abstract

Prurigo Strophulus is a common disease in Yaoundé (Cameroon), with a prevalence of 5.4%. Papule‐vesicle lesions are the most frequent signs. It is usually found in exposure areas of body.

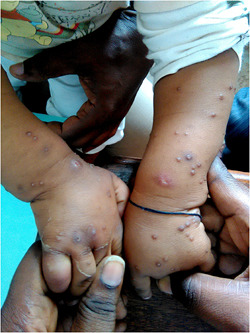

1

**What is already known about this topic?**

Prurigo strophulus (PS) is a recurrent itchy dermatosis in children secondary to arthropods bites. This dermatosis mainly affects children aged 2–7 years and those with atopic terrain and living in a poor social environment.
It is a frequent health situation/condition that can, over time, impact the quality of life of both patients and their families.

**What does this study add?**

Although frequently encountered, there are few documented studies on PS in our regions in general and in Cameroon in particular.
In Cameroon, the prevalence of PS was unknown and this study found a prevalence of PS of 5.4%.

## 2 INTRODUCTION

Prurigo strophulus (PS) is an inflammatory dermatosis related to a delayed hypersensitivity reaction to arthropod bites, occurring mainly in children.^1^ There are three clinical forms of PS: acute, subacute and chronic prurigo strophulus.^2^ In acute form, it is called prurigo simplex acuta or prurigo strophulus (PS); this dermatosis mainly affects children aged 2–7 years and those with atopic terrain^3^ and living in a low socio‐economic group.^4^ It is due to cellular hypersensitivity to environmental arthropods, and is characterized by pruritic, papular or papulovesicular erythematous skin lesions on the exposed areas, lower limbs, necking points (body stress points like elbow, shoulder) and trunk.^1^, ^4^, ^5^ The occurrence of PS seems to be linked to hot climate and poor socio‐economic conditions.^4^ Indeed, several cases have been described in Mexico, Thailand, Nigeria and Mali where the prevalence varies from 2.3 to 4.4%.^6^, ^7^, ^8^ To the best of our knowledge, in Cameroon, the prevalence of PS remains unknown. This is the reason why we carried out this study in order to determine the prevalence of PS and describe its clinical characteristics in hospitals in Yaoundé (Cameroon).

## 3 MATERIALS AND METHOD

### 3.1 Method

This was a descriptive cross‐sectional study that took place over a period of 3 months from February to May 2017 in six hospitals of Yaoundé, namely: Yaoundé Central Hospital (YCH), Biyem‐Assi District Hospital (BADH), Gyneco‐Obstetric and Pediatric Hospital of Yaoundé (GOPHY), University Teaching Hospital of Yaoundé (UTHY), Essos Hospital Center (EHC) and Subdivisional Health Center of Elig‐Essono. These hospitals were chosen by convenience based on the availability of dermatologist. Our sampling was non‐probabilistic and consecutive.

Our target population was children received in dermatological consultation for dermatosis during this period.

Were included in the study, children from 0 to 17 years old received in dermatological consultation in whom diagnosis of PS was made only by the dermatologist during physical exam and whose parents gave their consent.

Furthermore, all children and parents who refused to participate or who decided to withdraw were excluded from the study without any constraint (it did not affect their clinical care).

### 3.2 Procedures

#### 3.2.1 Ethical approval

We requested and obtained the research authorizations from the administrative authorities of the hospitals included in this study.

#### 3.2.2 Data collection

Data collection took place during dermatological consultations. Thus, after informing patients about the study and obtaining their informed consent, patients fulfilling the inclusion criteria were retained after the consultation. The questionnaire containing socio‐demographic data (age, sex, place of residence, type of housing, level of study), environmental data (presence of pets, carpets, double curtains and woollen mattress covers which expose to the occurrence of the PS. Indeed, arthropods usually hide in them) and clinical data (localization, papular‐vesicular lesions, bullous lesions, excoriated/ulcerated papules) were filled in by all the patients.

The diagnosis of PS was essentially clinically, based on young age associated with the presence of lesions (papules, papulo‐vesicles, papulo‐pustules) predominant in uncovered areas.

#### 3.2.3 Statistical analysis

The data collected were encoded in CSPRO 6.3 software, then exported to SPSS 23.0 for statistical analysis. The qualitative variables were described by their numbers and percentages and the quantitative variables by their means associated with their standard deviation.

### 3.3 Ethical considerations

Before the beginning of the study, an ethical clearance was obtained from the institutional ethics committee of the Faculty of Medicine and Biomedical Sciences of Yaoundé and research authorizations from the directors of hospitals choose for the study.

Patients were informed on the different aspects of the study and their agreement was only obtained after the parents gave their consent to participate to the study, and obtaining an agreement for children with age more than 7 years. We ensured the anonymity of the patients and the confidentiality of the information collected.

## 4 RESULTS

### 4.1 Prevalence of PS

During the study period, we received 2803 patients for dermatology consultation in the six selected health facilities. The diagnosis of PS was retained in 150 of these patients, meaning a prevalence of 5.4%. Thirty‐eight patients were adults and were excluded. Therefore, 112 children were included in the study.

### 4.2 Sociodemographic characteristics

We included a total of 112 predominantly male children (62/112; 55.4%), with a sex ratio of 1.2. The most represented age group was 0–5 years (78.6%) (Table 1). The median age was 2 years, with range from 5 months to 16 years.

**TABLE 1:** Socio‐demographic characteristics of the participants

Distribution of children Followed for PS		
Age (years)	*N*	Percentage (%)
0–5	88	78.6
6–10	13	11.6
11–15	9	8
16–20	2	1.8
Total	112	100
Level of education		
Non‐scholarized	80	71.4
Primary	17	15.2
Secondary	15	13.4
University	0	0
Total	112	100

Our study population consisted mainly of young children who were not scholarized. In these children, the median duration of PS was 8 months, with extremes of 1 and 108 months. The median time between the onset of the disease and the first dermatology consultation was 3.5 months with extremes of 1 and 96 months.

### 4.3 Environmental factors that may cause PS

We noted several environmental factors that could favour the occurrence of PS in our sample, in particular: the presence of pets (35/112; 31.3%), carpets (30/112; 26.8%), double curtains (4/112; 3.6%) and woollen mattress covers (1/112; 0.9%).

### 4.4 Clinical features

**FIGURE 1:**
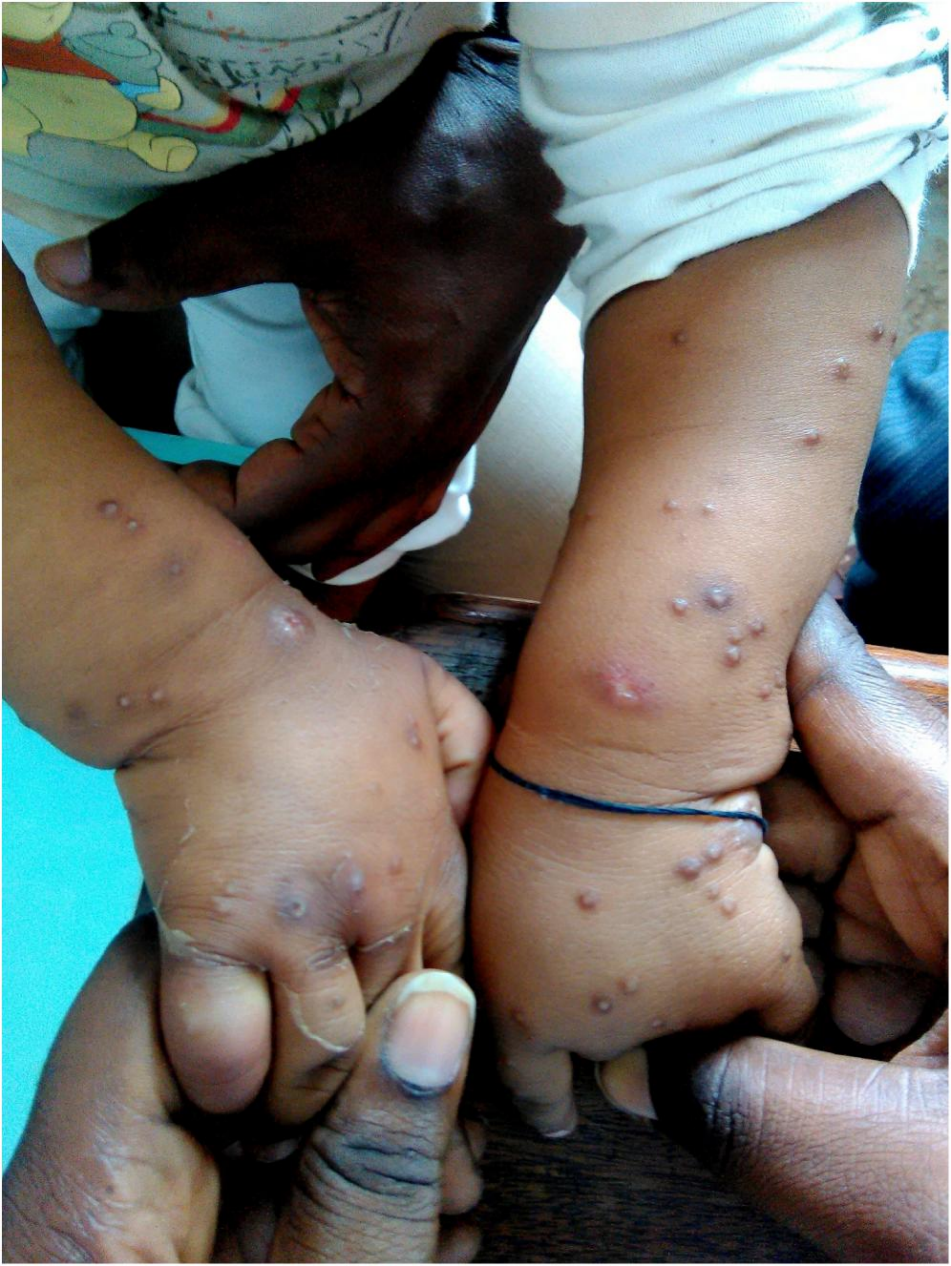
Prurigo strophulus lesions on the upper limbs in an infant

Lesions of PS were mainly located in the uncovered areas, in particular lower limbs (101/112; 90.2%) and upper limbs (85/112; 75.9%) (Figure 1), then the face (19.6%). The other seats were: buttocks (9.8%), back (6.3%), thorax (5.4%), neck (4.5%) and abdomen (2.7%).

The different types of lesions noted were grouped into two groups illustrated in Table 2. Papulo‐vesicular lesions (98/112; 87.5%) predominated in the primary lesion group and hypopigmented macular lesions with hyper‐pigmented halo in the group of secondary lesions (74/112; 66.1%).

**TABLE 2:** Different types of lesions found in children

Lesions	Type of Lesions	*N*	Percentage (%)
Primary	Papulo‐vesicle	98	87.5
	Urticarian papules	18	16.1
	Bulles	5	4.5
Secondary	Hypopigmentar macules with hyperpigmentar halo	74	66.1
	Excoriated/ulcerated papules	67	59.8
	Crusts	58	51.8
	Stratching lesion	15	13.4
	Impetiginisation	10	8.9
	Lichenification	6	5.4

## 5 DISCUSSION

### 5.1 Prevalence of PS

In this study, we found a prevalence of PS of 5.4% at the dermatological consultation, which is much higher than that of Mexican (2,2%) and Indian (3,6).^9^, ^10^ This high prevalence could be explained by the fact that in Yaoundé, climate is hot and humid with temperatures varying between 19.7 and 30.6°C during this study period (February–July) and its location at the heart of a forest area.

### 5.2 Sociodemographic characteristics of patients

The population in this study was mainly unscholarized (never been in education) children, with a median age of 2 years. The most represented age group was 0–5 years old (78.6%). This result is similar to those found in other hospital‐based studies, particularly in Ivory Coast, India and Congo where the most represented age group was 0–5 years old respectively,^4^ and 1–5 years.^10^, ^11^ The predominance of this age group could be explained by the immaturity of the immune system during childhood.^12^, ^13^

We found a male predominance in children (55.4%) with a sex ratio of 1.2. This result is close to the Indian study by Sardana et al.^10^ who had a sex ratio of 1. It is different from that found by Lenga et al.^11^ in Congo who reported a sex ratio of 0.4. During the same Congolese study, it was noted that 66% of the patients wearing uncovered clothing were girls. Unfortunately, we did not ask about uncovered clothes in our study as in Indian study. However, this corroborates the fact that girls do not often wear long‐sleeved clothes, unlike boys.

### 5.3 Environmental factors that may favour the onset of PS

We noted several environmental factors that could favour the occurrence of PS, including: the presence of pets (35/112; 31.3%), carpets (30/112; 26.8%), double curtains (4/112; 3.6%) and woollen mattress covers (1/112; 0.9%). Regarding the type of mattress, Halpert et al.^3^ in Nigeria found a statistically significant association between spring mattresses and the occurrence of PS (odds ratio [OR]: 1.84; 95% confidence interval CI [1.1–2.1]; *p* = 0.03). Arthropods are usually hidden in matelas, thick or lined curtains, carpets and armchairs, which may explain the significant associations found.

Patients should pay particular attention to their home environment, and find a way to eradicate athropods and insects from their immediate surroundings.

In this same Nigerian series, there were also a statistically significant association between the presence of fleas in households and the occurrence of PS (OR: 1.84; 95% CI [1.4–2.3]; *p* < 0.001).^3^ The other significantly associated factors were concerned with soil in the house, low socioeconomic level, history of atopic dermatitis in siblings (*p* < 0.05).^3^

### 5.4 Clinical characteristics of patients

The lesions of the PS were mainly located on the upper limbs (75.9%) and lower limbs (90.2%). These locations have been recognized in the literature.^1^, ^11^, ^14^ This preferential location in the exposed areas of the body makes the PS a condition difficult to conceal.

The papulo‐vesicle was the predominantly observed clinical lesion (87.5%). It was very often associated with other lesions, notably hypopigmented macule with hyper pigmented halo (66.1%) and excoriated/ulcerated papule (59.8%). These last two presentations bear witness to the tendency to scratching secondary to pruritus, which is the main symptom here.

### 5.5 Limits of the study

Our study may have limitations due to the fact that: (i) our study population was hospital‐based and therefore was not representative of the general population; and (ii) we have made a choice of convenience for the different hospitals where patient recruitment took place. Nevertheless, this study could constitute a basis for future study on a larger scale and sufficiently representative.

## 6 CONCLUSION

PS is a common condition in our environment, with a hospital prevalence of 5.4% in Yaoundé (Cameroon). The papulo‐vesicular form constitutes the main clinical presentation and located on uncovered body.

## CONFLICT OF INTERESTS

The authors declare that there are no conflict of interests.

## Data Availability

The data that support the findings of this study are available from the corresponding author upon request.

## References

[1] Philibert F , Baubion E , Amazan E , Ferrati‐Fidelin G . Recrudescence de prurigo strophulus en Martinique liée aux piqûres de culicoïdes. Ann Dermatol Venereol. 2019;146 (12):A244‐5.

[2] Akar HH , Tahan F , Balkanli S , Sadet Özcan S . Prurigo simplex subacuta or prurigo simplex acuta? Eur Ann Allergy Clin Immunol. 2014;46 (4):152–3. 25053633

[3] Halpert E , Borrero E , Ibañez‐Pinilla M , et al. Prevalence of papular urticaria caused by flea bites and associated factors in children 1‐6 years of age in Bogotá, D.C. World Allergy Org J. 2017;10 (1):36. 10.1186/s40413-017-0167-y PMC5674867 29158868

[4] Ahogo C , Sangare A , Yoboue P , et al. Prurigo strophulus : aspects épidémiologiques et étiologiques sur peau noire à Abidjan ‐ Côte d’Ivoire. Med Afr Noire. 2008;55 (6):313–6.

[5] Ássimos I , Pedrosa A , Martins P , Bettencourt H , Azevedo F . Bullous papular urticaria—case report and brief review of the literature. J Portuguese Soc Dermatol Venereol. 2012;70 (3):359. 10.29021/spdv.70.3.14

[6] Wisuthsarewong W , Viravan S . Analysis of skin diseases in a referral pediatric dermatology clinic in Thailand. J Med Assoc Thai. 2000;83 (9):999–1004. 11075964

[7] Yahya H . Change in pattern of skin disease in Kaduna, North‐Central Nigeria. Int J Dermatol. 2007;46 (9):936–43. 10.1111/j.1365-4632.2007.03218.x 17822496

[8] Mahé A , Cissé IA , Faye O , N′Diaye HT , Niamba P . Skin diseases in Bamako (Mali). Int J Dermatol. 1998;37 (9):673–6. 10.1046/j.1365-4362.1998.00454.x 9762817

[9] Del Pozzo‐Magaña BR , Lazo‐Langner A , Gutiérrez‐Castrellón P , Ruiz‐Maldonado R . Common dermatoses in children referred to a specialized pediatric dermatology service in Mexico: a comparative study between two decades. ISRN Dermatol. 2012;2012:1–5. 10.5402/2012/351603 PMC3477666 23097714

[10] Sardana K , Mahajan S , Sarkar R , Mendiratta V , Bhushan P , Koranne RV , et al. The spectrum of skin disease among Indian children. Pediatr Dermatol. 2009;26 (1):6–13. 19250398 10.1111/j.1525-1470.2008.00814.x

[11] Lenga A , Lenga‐Loumingou I , Moussounda M , Vouidibio J . The Prurigo Strophulus in Brazzaville: demonstration of vectors and study of some associated bioecological parameters. Pak J Zool. 2013;45:121–8.

[12] Ayanlowo O , Puddicombe O , Gold‐Olufadi S . Pattern of skin diseases amongst children attending a dermatology clinic in Lagos, Nigeria. Pan African Med J. 2018;29 (1):1–10. 10.11604/pamj.2018.29.162.14503 PMC6057566 30050626

[13] Thummanapally N , Lawdyavath K , Guruva C , Enumula D , Pvk S , Anchuri SS . Prevalence of childhood skin disorders attending at outpatient pediatric hospital. Asian J Pharm Clin Res. 2020:131–5. 10.22159/ajpcr.2020.v13i5.35578

[14] Raza N , Lodhi MS , Ahmed S , Dar NR , Ali L . Clinical study of papular urticaria. J Coll Phys Surg Pak. 2008;18 (3):147–50. 18460241

